# Modified Pedicle Grafting: A Novel Noninvasive Technique for Soft Tissue Augmentation Around Maxillary Dental Implants

**Published:** 2018-01

**Authors:** Seyed Hossein Mohseni Salehi, Afshin Khorsand, Sahar Chokami Rafiei, Faris Yousif Mirkhan

**Affiliations:** 1 Assistant Professor, Department of Periodontics, School of Dentistry, Tehran University of Medical Sciences, Tehran, Iran; 2 Associate Professor, Department of Periodontics, School of Dentistry, Tehran University of Medical Sciences, Tehran, Iran; 3 Assistant Professor, Department of Periodontics, School of Dentistry, Shahid Sadoughi University of Medical Sciences, Yazd, Iran; 4 Postgraduate Student, Department of Periodontics, School of Dentistry, International Campus, Tehran University of Medical Sciences, Tehran, Iran

**Keywords:** Maxillary, Dental Implants, Surgical Flaps, Soft Tissue, Augmentation, Vestibuloplasty

## Abstract

**Objectives:**

This study sought to assess the efficacy of modified pedicle grafting as a noninvasive technique for soft tissue augmentation around maxillary dental implants.

**Materials and Methods:**

This descriptive study was conducted on eight patients who met the inclusion criteria. Prior to the second-stage surgery for exposing the implants, the buccal keratinized mucosa width, vestibular depth, and mucosal thickness around the implants were measured. The same parameters were measured six months after the second-stage surgery and were compared with the baseline values. Also, the color match of the graft with the adjacent gingival and mucosal tissues was evaluated.

**Results:**

Forty-seven maxillary implants were evaluated. The minimum and maximum gains of keratinized mucosal width were respectively equal to 0mm and 7mm, with a mean of 4.31±1.19mm. The mean vestibular depth around the implants was 9.47±1.75mm (ranging from 5mm to 12mm) six months after the surgery. At the beginning of the study, a thin mucosa surrounded the implants, but after six months, the peri-implant keratinized mucosa width increased. The color match of the graft with the adjacent gingival and mucosal tissues was excellent based on the periodontists’ opinion.

**Conclusions:**

Modified pedicle grafting is a safe and predictable technique for soft tissue augmentation around maxillary implants. This technique is reliable for increasing the width of keratinized mucosa in fully and partially edentulous patients with a shallow vestibular depth. The stability of the pedicle flap is achieved by fixing the flap to the tissue around the healing abutment.

## INTRODUCTION

The high success rate of dental implant treatment has been well-documented. Adherence to the principles of implant osseointegration minimizes the rate of complications. The health and stability of peri-implant tissues guarantee the long-term success of dental implants [1].

The role of keratinized mucosa in the survival of intraosseous dental implants is still a matter of debate [2]. The absence of keratinized mucosa complicates a proper oral hygiene maintenance. Evidence shows a correlation between the width of keratinized mucosa and plaque accumulation and gingival inflammation [3].

Up to 50% of the primary width of the bone may be lost after tooth extraction; as a result, the mucogingival line will be coronally displaced. This may necessitate one or two additional surgical procedures for dental implant placement [4]. In many cases, the clinician may encounter an insufficient attached gingiva or decreased vestibular depth for surgical wound closure in severely resorbed maxillary ridges or after flap advancement during bone augmentation surgery. Several techniques have been suggested for increasing the width of the keratinized mucosa around dental implants such as the apically positioned flap (APF), pedicle graft, connective tissue graft (CTG), and free gingival graft (FGG) by use of allografts. The pedicle grafting technique has some advantages such as suitable vascularization; however, this technique is more time-consuming than the other grafting techniques [5]. This study aimed to assess the efficacy of a palatal pedicle graft for increasing the keratinized mucosa width around dental implants.

## MATERIALS AND METHODS

This descriptive study was conducted on the patients presenting to the Implant and Periodontics Departments of School of Dentistry of Tehran University of Medical Sciences, who met the study’s inclusion criteria. The patients were selected by convenience sampling and were systemically healthy and had several dental implants in the maxilla with a maximum of 2mm or no keratinized mucosa at the buccal surfaces of the implants.

**Fig. 1:**
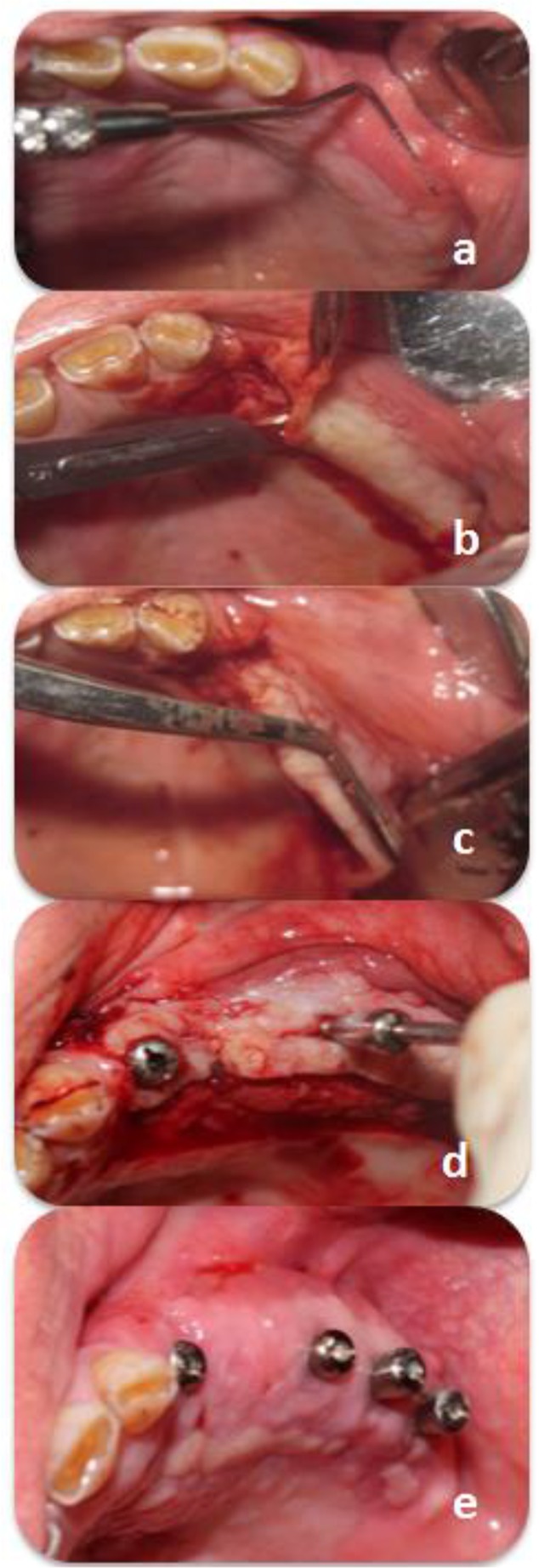
(a) The roll test. (b) The first incision. (c) The split-thickness flap. (d) A small incision, 3mm far from the flap edge. (e) Sufficient keratinized mucosa width and vestibular depth after 30 days

The patients were examined immediately before the second-stage surgery (the baseline). The following clinical parameters were recorded six months after the second-stage surgery and were compared to the baseline values:

1. The width of the keratinized mucosa at the buccal surfaces of the implants was determined using the rolling technique for identifying the mucogingival junction (Fig 1. a). The shrinkage of the graft was measured according to the following formula:
  $$\text{Shrinkage}=\frac{\begin{array}{l} \text{difference}\;\text{in}\;\text{the}\;\text{mean}\;\text{width}\;\text{of}\;\text{keratinized}\;\text{mucosa}\;\left(\text{mm}\right)\\ \text{at}\;\text{the}\;\text{baseline}\;\left(5\text{mm}\right)\;\text{and}\;\text{six}\;\text{months}\;\text{after}\;\text{the}\;\text{second}\;\text{surgery} \end{array}}{\text{Width}\;\text{of}\;\text{keratinized}\;\text{mucosa}\;\left(\text{mm}\right)\;\text{at}\;\text{the}\;\text{baseline}\;\left(5\text{mm}\right)\;}\times 100$$
2. The thickness of the keratinized mucosa at the buccal surfaces of the implants was determined by placing a probe in the sulcus. The keratinized mucosa was considered to be thin if the shadow of the probe could be seen through the mucosa and thick if the shadow could not be seen [6,7].
3. The vestibular depth was measured from the crest of the alveolar ridge to the bottom of the vestibule by using a probe. If this value was less than 4mm, the vestibular depth would be considered shallow. If this value was more than 4mm, the vestibular depth would be considered adequate [8].
4. To assess the color match of the graft with the adjacent gingival and mucosal tissues, photographs were taken of the area by a digital camera (Canon Inc., Tokyo, Japan) under standard conditions. The photographs were viewed by three periodontists, and they expressed their opinions in terms of the color match. The University of Michigan “O” probe with William’s grading (1, 2, 3, 5, 7, 8, 9, and 10mm) was used for the clinical measurements.

### Surgical technique:

Local infiltration anesthesia was administered using 2% lidocaine with 1:100,000 vasoconstrictor (Daru-Pakhsh Pharmaceutical Mfg. Co., Tehran, Iran). During the surgical phase, a palatal pedicle graft [9] was designed for uncovering the implants and placing the healing abutments. An 8-mm wide trapezoidal split-thickness flap (at a distance of 8mm from the mucogingival line) was elevated and positioned buccally such that the flap tip was placed 3mm palatal to the implants (Fig 1. b–d).

A small incision, parallel to the primary incisions, was made at the neck of the healing abutment. The healing abutment was connected to the submerged implant via this incision, and the flap was fixed in place. The flap was also fixed to the healing abutment using continuous sling sutures.

To ensure the apical stability of the flap, several additional sutures were made at the depth of the vestibule by using violet polyglycolic acid (PGA)-coated synthetic absorbable suture (Supabon, SUPA Medical Devices Co., Tehran, Iran). After suturing, the surgical wound was covered by Coe-Pak™ surgical dressing (GC America Inc., Alsip, IL, USA).

The patients were prescribed with 500mg *amoxicillin*, three times a day for 10 days and 0.12% chlorhexidine mouthwash, twice a day for four weeks. The sutures were removed 14 days after the surgery. Photographs were taken of the area 30 days after the surgery (Fig. 1. e).

### Sample size determination and statistical analysis:

The sample size was predicted based on the descriptive studies’ formula. According to the study by Gilbert et al [10], by using the option of comparing the two means in the Minitab software (Minitab Inc., Pennsylvania, USA) and by considering standard deviation (SD)=0.85 and minimum accuracy=0.5mm, the minimum sample size was estimated to be 18 samples. Statistical analysis was performed by using SPSS® version 22 software program (SPSS for Windows, IBM Co., Chicago, IL, USA).

## RESULTS

Eight patients, including 4 males and 4 females, with the mean age of 51.5±13.18 years (28 to 66 years old) were evaluated. Table 1 shows the number of the implants in male and female patients.

**Table 1.** Age, gender, and number of implants of the participants

**Patient**	**Age (years)**	**Gender**	**Number of implants**
1	28	Female	8
2	58	Female	3
3	66	Male	4
4	52	Male	8
5	38	Female	8
6	49	Female	4
7	55	Male	4
8	62	Male	8
Mean	51.50		5.87

### Implant distribution:

Forty-seven maxillary implants were evaluated (Table 1). Table 2 shows the distribution of the implants.

**Table 2.** Frequency distribution and percentage of the implants placed in different oral regions

**Site**	**Number of implants**	**Percentage**
Central incisor	3	6.4
Canine	9	19.4
First premolar	9	19.4
Second premolar	12	25.5
First molar	8	17
Second molar	6	12.8

The width of keratinized mucosa ranged between 0–2mm (mean=0.17±0.43mm) before the surgical procedure. No keratinized mucosa was detected around 85% of the implants (n=40).

Six months after the surgery, the width of keratinized mucosa was greater than 5mm around 50% of the implants. The mean width of the keratinized mucosa around the implants was 4.49±1.81mm, with a maximum width of 7mm and a minimum width of 1mm (Table 3).

**Table 3.** Keratinized mucosa width (mm) and vestibular depth (mm) around the implants at the baseline and six months after the surgery

**Variable**	**Minimum**	**Maximum**	**Mean**	**SD**
Preoperative keratinized mucosa width	0	2	0.17	0.433
Keratinized mucosa width after 6 months	1	7	4.49	1.081
Preoperative vestibular depth	2	2	2.00	0.000
Vestibular depth after 6 months	5	12	9.47	1.755

SD=Standard Deviation

### Vestibular depth:

Since the flap had been advanced (for wound closure following guided bone regeneration), a great coronal (palatal) displacement of the mucogingival junction had occurred.

The vestibular depth was shallow in all patients (less than 4mm). The mean vestibular depth around the implants at the baseline was considered to be 2mm. The mean vestibular depth around the implants was 9.47±1.75mm (ranging from 5mm to 12mm) six months after the surgery (Table 3).

The changes in the vestibular depth and keratinized mucosa width around the implants after six months were statistically significant compared to the baseline (P<0.05). The keratinized mucosa gain and vestibular depth gain were significantly correlated (P<0.001, Table 4).

**Table 4.** Keratinized mucosa gain (mm) and vestibular depth gain (mm)

	**Number of implants**	**Minimum**	**Maximum**	**Mean**	**SD**	P-value^*^
**Keratinized mucosa gain**	47	0.00	7.00	4.3191	1.19975	P< 0.001
**Vestibular depth gain**	47	3.00	10.00	7.4681	1.75513	P< 0.001

* According to t-test in comparing the measurements before and 6 months after the surgery, SD=Standard Deviation

The peri-implant mucosa was thin at the beginning of the study, but six months after the surgery, a thick keratinized mucosa was detected around the implants. The color match of the graft with the adjacent gingival and mucosal tissues was excellent based on the periodontists’ opinion. Six months after the surgery, the shrinkage of the modified pedicle graft was equal to 13.8%.

## DISCUSSION

A poor oral hygiene and the characteristics of the peri-implant tissues such as the absence of the cementum and periodontal ligament (PDL), inadequate blood vessels, scarce fibroblasts, parallel orientation of the supracrestal connective tissue fibers, and subgingival position of crown margins, which are different from the characteristics of the tissues around natural teeth, make the implants susceptible to inflammation and bone loss in case of plaque accumulation and microbial invasion. Thus, failure to establish a peri-implant biological seal can significantly affect the implant’s survival [2].

Dental implants surrounded by less than 2mm of keratinized mucosa are highly susceptible to gingival recession and alveolar bone loss. In the absence of keratinized mucosa, plaque accumulation is significantly increased [11,12]. In this study, we assessed the efficacy of modified pedicle grafting as a novel and noninvasive technique for soft tissue augmentation around maxillary implants.

In a study on eight patients, Elkhaweldi et al [1] performed an APF in one patient prior to implant placement at two sites. Three months later, the keratinized mucosa width was increased by 5mm. The reason for a greater keratinized mucosa gain in the study by Elkhaweldi et al [1] compared to ours (5mm versus 4.3mm) may be attributed to a shorter duration of follow-up (three months after the surgery versus six months) and conduction of the surgery before implant placement (a higher blood supply due to the absence of the implant). Moreover, they only performed the APF at two sites. Our results are more reliable because of the larger sample size. Three months after the second-stage surgery, the peri-implant keratinized mucosa width was increased by 3.1mm in three patients at six areas subjected to the APF [1]. Both techniques (pedicle flap and APF) provide a suitable blood supply for the flap. The significant keratinized mucosa gain in our study was due to the placement of a 5-mm wide keratinized mucosa at the buccal surfaces of the implants by modified pedicle grafting, which resulted in a greater width of keratinized mucosa around the implants. In the study by Elkhaweldi et al [1], a pedicle flap was performed in one patient during the second-stage surgery. After three months, a 2.4-mm keratinized mucosa width was achieved. This technique is similar to the modified pedicle flap technique. In the mentioned study, the keratinized mucosa gain after three months was equal to 2-3mm in one patient following the use of an FGG in two areas [1], which was less than the gain in our study. The reason may be attributed to the use of an FGG. After the use of a CTG, no gain in the keratinized mucosa width was noted around the implants [1], which may be due to the absence of keratinized mucosa around the implants at the beginning of the study. A previous study reported 1.4mm of keratinized mucosa gain following the use of a dermal matrix graft [13]. This value was equal to 2.4mm after the use of autogenous grafts or subepithelial CTGs [13]. The increase in the keratinized mucosa width was equal to 2.5mm after the use of an FGG, which yielded the highest keratinized mucosa gain compared to other techniques [13]. In modified pedicle grafting, which involves noninvasive soft tissue augmentation around maxillary implants, the keratinized mucosa width increased by 4.3mm, which was approximately two times higher than the gain following the use of other techniques. The rate of the keratinized mucosa shrinkage following the use of autogenous grafts is higher than 40% [8], while the pedicle flap showed the least amount of shrinkage during the present study. In a retrospective assessment of patients, the rate of the shrinkage of keratinized mucosa after the use of an autogenous FGG was 38% to 45% during six months [1]. In a systematic review, the shrinkage rate of keratinized mucosa following an autogenous FGG in the control group was 32.92% after six months. This value was 41.12% in the test group six months after the use of a xenograft [13]. In the current study, the shrinkage rate of the modified pedicle graft was 13.8% after six months, which was lower than the value reported in previous studies and can be attributed to the proper blood supply provided by the pedicle flap and less trauma to the flap. Gaining 4.3mm of peri-implant keratinized mucosa enhances plaque control and resistance to masticatory forces and provides optimal aesthetics and phonetics. The noninvasive soft tissue augmentation technique used in the present study caused an increase in the thickness of the keratinized mucosa around the maxillary implants. In this study, the vestibular depth increased by a minimum of 5mm and a maximum of 12mm six months after performing a modified pedicle flap around maxillary implants. The mean gain of vestibular depth was 7.46mm in the present study. A significant direct correlation was noted between the amount of keratinized mucosa and vestibular depth such that a greater amount of keratinized mucosa was associated with a greater vestibular depth. A 1-mm increase in the keratinized mucosa gain yielded a 1.73-mm *increase in the vestibular depth:*

$$\frac{\mathrm{Mean}\;\mathrm{vestibular}\;\mathrm{depth}\;\mathrm{gain}}{\mathrm{Mean}\;\mathrm{keratinized}\;\mathrm{mucosa}\;\mathrm{gain}}=\frac{7.46\text{mm}}{4.31\text{mm}}=1.73\mathrm{mm}$$

Regarding the color match of the grafted peri-implant keratinized mucosa with the surrounding gingiva, the photographs showed an excellent color match six months after the surgery. At the onset of the study, the mucosa around the implants was thin such that the shadow of the periodontal probe could be seen through the mucosa; however, the mucosal thickness around the implants increased after six months such that the shadow of the periodontal probe could no longer be seen through the mucosa. A small sample size and short duration of follow-up (six months) were among the limitations of the present study.

## CONCLUSION

The novel technique of pedicle grafting around maxillary implants is a safe and predictable method for soft tissue augmentation. This technique is reliable for increasing the width of keratinized mucosa in fully and partially edentulous patients with a shallow vestibular depth. The stability of the pedicle flap is an advantage of this technique, which is achieved through fixing the flap to the tissue around the healing abutment.
